# Circulating Exosomal circRNAs Contribute to Potential Diagnostic Value of Large Artery Atherosclerotic Stroke

**DOI:** 10.3389/fimmu.2021.830018

**Published:** 2022-01-13

**Authors:** Qi Xiao, Rongyao Hou, Hong Li, Shuai Zhang, Fuzhi Zhang, Xiaoyan Zhu, Xudong Pan

**Affiliations:** ^1^ Department of Neurology, The Affiliated Hospital of Qingdao University, Qingdao, China; ^2^ Department of Neurology, The Affiliated Hiser Hospital of Qingdao University, Qingdao, China; ^3^ Department of Critical Care Medicine, The Affiliated Hospital of Qingdao University, Qingdao, China

**Keywords:** large artery atherosclerotic stroke, exosome, circRNAs, atherosclerosis, biomarkers, immune cells

## Abstract

Large artery atherosclerotic (LAA) stroke is closely associated with atherosclerosis, characterized by the accumulation of immune cells. Early recognition of LAA stroke is crucial. Circulating exosomal circRNAs profiling represents a promising, noninvasive approach for the detection of LAA stroke. Exosomal circRNA sequencing was used to identify differentially expressed circRNAs between LAA stroke and normal controls. From a further validation stage, the results were validated using RT-qPCR. We then built logistic regression models of exosomal circRNAs based on a large replication stage, and receiver operating characteristic (ROC) curves were constructed to assess the diagnostic efficacy. Using exosomal circRNA sequencing, large sample validation, and diagnostic model construction revealed that exosomal circ_0043837 and circ_ 0001801were independent predictive factors for LAA stroke, and had better diagnostic efficacy than plasma circRNAs. In the atherosclerotic group (AS), we developed a nomogram for clinical use that integrated the two-circRNA-based risk factors to predict which patients might have the risk of plaque rupture. Circulating exosomal circRNAs profiling identifies novel predictive biomarkers for the LAA stroke and plaque rupture, with superior diagnostic value than plasma circRNAs. It might facilitate the prevention and better management of this disease.

## Introduction

Stroke is a global public health problem. According to the Global Burden of Disease Study 2019, stroke is the third leading cause of death and disability (1). Approximately 80% of strokes are ischemic, of which, the large artery atherosclerotic (LAA) stroke is an important subtype (2). LAA stroke is closely related to atherosclerosis, in which plaque rupture and thrombosis are key causes of disease onset and progression (3, 4), associated with the accumulation of immune cells in the vascular intima (5). Therefore, it is important to develop potential approaches for the diagnosis of LAA stroke and plaque rupture.

Circular RNAs (circRNAs), a type of non-coding RNAs, are candidate biomarkers and potential therapeutic targets due to their tissue specificity (6, 7). Furthermore, they play an essential role in the pathophysiology of ischemic stroke and atherosclerosis. Studies have reported that the presence of circulating circRNAs may potentially aid in the diagnosis of acute ischemic stroke (8). In atherosclerosis pathogenesis, circRNAs are involved in the apoptosis of immune cells (macrophages), migration of vascular smooth muscle cells; and formation of new intima (9, 10). However, circulating circRNAs are susceptible to degradation by biological enzymes and limitations of origin identification. Nonetheless, recent studies have shown that circRNAs are stable and present in large amounts in exosomes (11, 12).

Exosomes are endogenous vesicles (approximately 40–160 nm in diameter) and can carry circRNAs and other biomolecules (13). Thus, exosomes can play critical roles in material transport, cell communication, and targeted therapy; moreover, they protect circRNAs from biological enzymes (14). Exosomes are secreted from various cell types with membrane specificity and targeting properties (15). Furthermore, researchers have identified exosomes involved in the atherosclerotic process (16). These included the endothelium-derived exosomes carrying miRNAs, which could inhibit macrophage infiltration and regulate the atherosclerotic plaque area (17). Our previous study also found that miRNA-145 present in the umbilical cord stem cell-derived exosomes reduced atherosclerotic plaques (18). Thus, exosomes could also serve as carriers of circRNAs, which may have diagnostic value for LAA stroke.

Therefore, in this study, we aimed to identify potential diagnostic and predictive biomarkers for LAA stroke and plaque rupture through exosomal circRNA sequencing and validation, that would facilitate immediate and appropriate management and prevention of LAA stroke.

## Materials and Methods

### Study Population

A total of 621 participants were enrolled in the study between June 2018 and December 2020 at the Affiliated Hospital of Qingdao University. Of the 621 participants, 366 were included in the acute ischemic stroke group (large artery atherosclerosis [LAA] group: 196 cases; small artery occlusion [SAO] group: 170 cases), 106 patients in the acute stroke (AS) group, and 149 participants in the normal control (NC) group.

The inclusion criteria for the acute ischemic stroke group were as follows (1): a diagnosis of an acute ischemic stroke using cranial CT or MRI (2); a diagnosis within 3 days of symptom onset (3); both LAA and SAO subtypes were included according to the Trial of Org 10172 in Acute Stroke Treatment (TOAST) criteria (19); and (4) those who did not undergo thrombolytic therapy. The inclusion criteria for the AS group were as follows (1): computed tomography angiography (CTA) of the head and neck assisted identification of intracranial or extracranial vascular stenosis > 50% or occlusion and (2) Exclusion of possibility of stroke by cranial CT or MRI. Healthy controls were recruited at the Medical Examination Center of the Affiliated Hospital of Qingdao University (2, 8, 20).

The exclusion criteria were as follows (1): other types of stroke such as hemorrhage, craniocerebral trauma, tumors, and neurological diseases (2); atrial fibrillation, myocardial infarction, arteritis, or any circulatory disease (3); severe pulmonary, hepatic, or renal dysfunction; and (4) malignant tumors, severe infection, autoimmune diseases, and other systemic diseases (2, 8, 20).

### Specimen Collection and Exosome Extraction

Fasting venous blood samples were collected in ethylenediamine tetra acetic acid (EDTA) tubes early in the morning within 24 h of admission, stored at 4°C for 30 min, and centrifuged at 2500 × *g* for 15 min. The upper layer of plasma was collected and stored at -80°C until further use.

Plasma exosomes were extracted using the Exosome Extraction Kit (Invitrogen, Cat 4484450, Carlsbad, USA) (21). Following the manufacturer’s instructions, the plasma was centrifuged at 2000 × *g* and 10,000 × *g* for 20 min at 25°C to remove impurities. Approximately 0.5 volume of phosphate buffer saline (PBS) and 0.05 volume of proteinase K were added, and the mixture was incubated at 37°C for 10 min. A total of 0.3 volume of exosome extraction reagent was added, and the mixture was incubated at 4°C for 30 min and centrifuged at 10,000 × *g* for 5 min. The creamy white precipitate of exosomes, visible at the bottom or on the wall of the tube, was resuspended in PBS.

### Electron Microscopy of Exosome Identification

The morphology of the extracted exosome was evaluated using an electron microscope. Briefly, the resuspended exosome solution (10 µL) was placed on a copper grid for 2 min at room temperature. Next, 2% phosphotungstic acid was added onto exosomes after which they were washed with distilled water. Finally, they were evaluated under the electron microscope (Hitachi, Tokyo, Japan) (22, 23).

### Identification of Exosome Particle Size

The diameter of the extracted exosomes was determined by measuring the particle size. Resuspended exosome solutions were analyzed using the ZetaView inspection instrument (Particle Metrix, Meerbusch, Germany) and the Network Traffic Analysis (NTA) software (ZetaView 8.04.02) to determine the size and number of exosome particles (22, 23).

### Western Blot Analysis for Exosome Identification

Exosome-specific markers, including positive (CD9, CD63, and TSG101) and negative markers (GRP94), were used to identify exosomes by western blotting. Total proteins (25 µg) in the extracted resuspension of exosomes were sequentially subjected to gel electrophoresis (10% SDS-PAGE), membrane transfer, blocking, incubation with primary antibodies specific for CD9, CD63, TSG101, and GRP94 (ab92726, ab134045, ab125011, ab238126, Abcam, Cambs, UK), incubation with the goat anti rabbit secondary antibodies, and enhanced chemiluminescence (ECL) to examine exosomal protein expression (22, 23).

### Sequencing of Exosomal CircRNA

Sequencing of exosomal circRNA was performed sequentially through sample collection and preparation; RNA extraction; and qualitative and quantitative analysis of extracted RNA purity, concentration, and integrity. Library quality control were performed on the Agilent Bioanalyzer 2100 system. The library was sequenced using the Illumina HiSeq 4000 platform.

Bioinformatic analysis of circRNA sequencing was performed by quality control, and circRNAs were detected and identified using find_circ and CIRI2 (24). Raw counts were normalized using the TPM (25).

### Gene Ontology and Kyoto Encyclopedia of Genes and Genomes Functional Enrichment Analysis

We performed the GO functional enrichment of the source genes of differentially expressed circRNAs using the GOseq R package, and GO terms with corrected *P* values < 0.05 were considered significantly enriched for differentially expressed genes. The KO-Based Annotation System (KOBAS) software was used to detect differentially expressed circRNA-derived genes enriched in the KEGG pathway (26).

### Reverse Transcription for Real-Time Quantification

Total RNA was extracted from plasma and resuspended exosomes using the miRNeasy^®^ Mini kit (Qiagen, DUS, Germany). Reverse transcription and quantitative amplification were performed sequentially using the RT6 cDNA Synthesis Kit, version 2 (TsingKe, Beijing, China) and the T5 Fast qPCR Mix (SYBR Green I) (TsingKe, Beijing, China). Amplification specificity was calibrated by generating a melting curve. *ACTB* was selected as the internal reference gene. The fold change in circRNA expression was calculated using the following formula:


$$2^{-\Delta \Delta Ct}$$

The primer sequences used in this study are listed in **Supplemental Table 1**.

### Statistical Analyses

Categorical variables were expressed as percentages and analyzed using the χ^2^ test. Continuous variables were expressed as mean ± SEM for those with a normal distribution or interquartile for those with a skewed distribution. Continuous variables were calculated through analysis of variance (ANOVA), *t*-test, and Kruskal-Wallis test. The Spearman test was used to calculate component correlations.

The potential factors were sequentially screened by univariate and multivariate binary logistic regression analysis. From among the set of variables, select those variables with P < 0.05 in the multivariate regression to include in the prediction model (27). The Hosmer-Lemeshow goodness-of-fit test was used to evaluate the calibration degree of the prediction model. The receiver operating characteristic (ROC) curve was used to evaluate the discrimination ability of the prediction model. Nomogram was done with the rms package of R software ver.4.1.1 (28). All analyses were performed using the Statistical Package for the Social Sciences (SPSS), ver. 26.0 (IBM Corp. in Armonk, NY) and the statistical significance was set at *P* < 0.05.

## Results

### The Landscape of Circulating Exosomal CircRNAs

**Figure 1:**
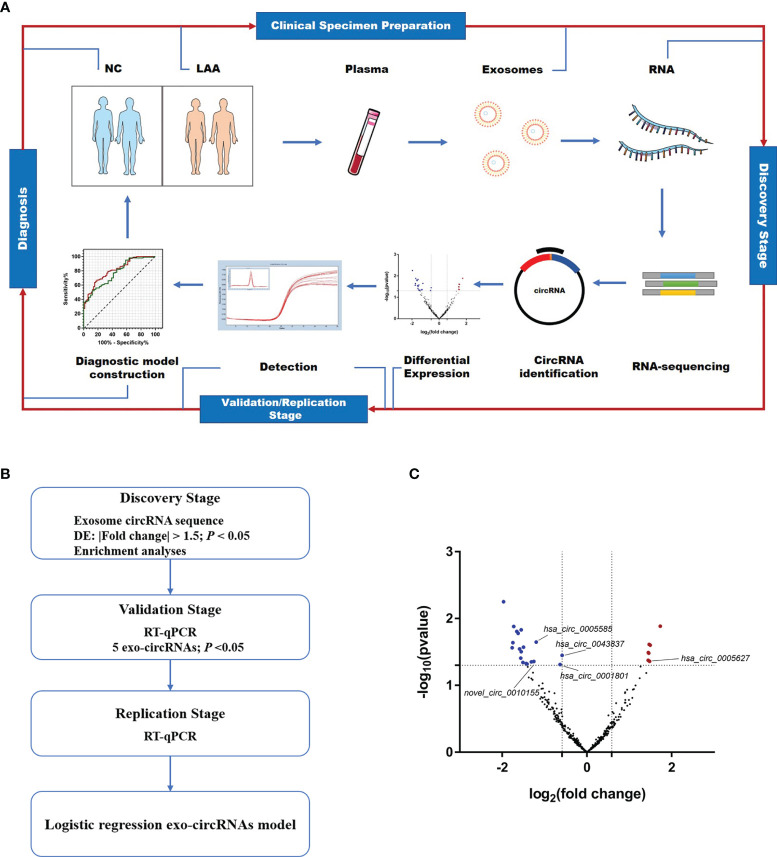
Expression profiles of circulating exosome-circRNAs. **(A)** Workflow of the study, including clinical samples preparation, discovery stage of circRNA sequencing, detection and diagnosis. **(B)** Workflow of the discovery, validation and replication stage of circulating exo-circRNAs. **(C)** Volcano map of circRNAs in exosome RNA-seq, with red showing up-regulated expression in the LAA group and blue showing down-regulation.

To better understand the potential role of exosomal circRNAs in the diagnosis of LAA stroke, we included 366 patients with new cerebral infarction and classified them according to TOAST (19). There were 196 patients with LAA stroke, 170 with SAO stroke, and 149 who were normal controls (**Supplemental Table 2**). As shown in **Figure 1A**, we performed circulating exosome transcriptome sequencing to determine differentially expressed circRNAs for further validation and identify possible biomarkers through diagnostic model construction.

We extracted circulating exosomes from the plasma and verified their quantity and integrity according to the standards of the International Society for Extracellular Vesicles (29). As shown in **Supplemental Figure 1**, the extracted circulating exosomes underwent morphological blood identification using electron microscopy, which showed that the exosomes appeared to be oval without a nucleus. NTA measurements showed that the diameter of exosomes ranged from 50–150 nm. Further, the markers enriched in exosomes were detected using western blotting. The positive markers were positive in the exosome group and negative in the residual supernatant. The negative markers were not detected in the exosomes. In summary, the morphology, number, and integrity of exosomes in the enriched exosome samples were assessed using the aforementioned methods.

As shown in **Figure 1B**, a workflow of the discovery, validation and replication stage of circulating exo-circRNAs. In the discovery stage, we performed exosomal circRNA sequencing of LAA stroke. We identified differentially expressed (DE) circRNAs by comparing the two groups bioinformatically. A total of 26 DE circRNAs were identified in NC and LAA subjects (*P* < 0.05, |fold change| ≥ 1.5). There were 7 significantly upregulated and 19 significantly downregulated genes in the LAA group compared to the NC group (**Figure 1C**).

To determine the function of DE circRNAs, we performed functional enrichment of the source genes of DE circRNAs, including the GO and KEGG pathway enrichment. The KEGG pathway showed that DE circRNAs could be enriched in antigen processing and presentation, classical signaling pathways, such as ERK, NF-kappa B and mTOR (**Supplemental Figure 2A**). We found that their biological processes mainly included immune, inflammatory, and metabolic pathways circRNAs using the GO database. Other cellular components in the cell membrane such as vesicle membrane and other component sources were also elucidated (**Supplemental Figure 2B**).

### Exosomes Derived CircRNAs as Biomarkers of LAA Stroke

**Figure 2:**
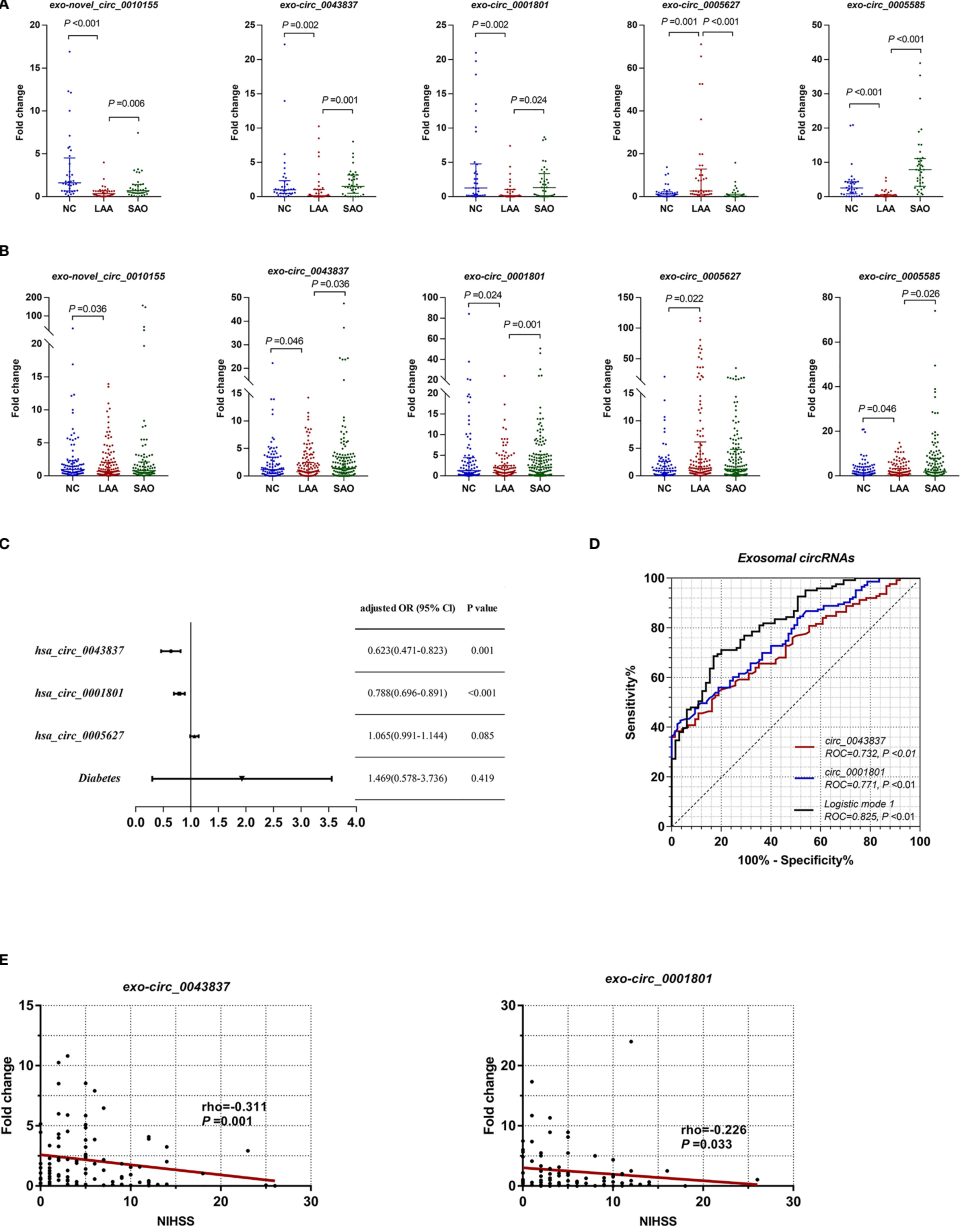
Expression of exosomal circRNAs as novel biomarkers for LAA stroke. **(A)** Validation of exosomal novel_circ_0010155, circ_0043837, circ_0001801, circ_0005627, and circ_0005585 in NC, LAA, and SAO groups. **(B)** Replication of exosomal novel_circ_0010155, circ_0043837, circ_0001801, circ_0005627, and circ_0005585 in the NC, LAA, and SAO groups**. (C)** Multivariate logistic regression analysis for exo-circRNAs. **(D)** ROC curves of the exosomal circRNAs, logistic mode 1: Two-exo-circRNA of hsa_circ_0043837 and hsa_circ_0001801. **(E)** Comparison between exosome derived circRNA and NIHSS score.

Combining the differential expression and functional enrichment of immune biological process, we further selected circRNAs that might be involved in the LAA process for small sample RT-qPCR validation. We selected the following: novel_circ_0010155, circ_0043837, circ_0001801, circ_0005627, and circ_0005585. As shown in **Figure 2A**, novel_circ_0010155, circ_0043837, circ_0001801, and circ_0005585 were significantly downregulated in the LAA group and were statistically different from the SAO group. circ_0005627 was significantly upregulated in the LAA group, while the expression of the SAO and NC groups was lower than that of the LAA group.

Based on the validation of small samples, we further performed a replication stage, in which we included 150 cases of LAA, 130 cases of SAO, and 103 NCs. As shown in **Figure 2B**, there were statistically significant differences in novel_circ_0010155, circ_0043837, circ_0001801, circ_0005627, and circ_0005585 compared with NCs. Of these, the expressions of novel_circ_0010155, circ_0043837, circ_0001801, and circ_0005585 were significantly downregulated, while that of circ_0005627 was significantly upregulated in the LAA group compared with the NC group. This had the same trend as that of the results of the small sample validation. Furthermore, circ_0043837, circ_0001801 and circ_0005585 showed statistically different expression levels between the LAA and SAO groups. However, novel_circ_0010155 and circ_0005627 was not statistically different among the two groups in the large sample replication.

To confirm the diagnostic value of five circRNAs for LAA stroke, we constructed the LAA diagnostic model through the logistic regression. We included five circRNAs and five clinical indicators (Smoking, drinking, hypertension, diabetes, and LDL) for binary logistic regression analysis. After multivariable adjustment by three circRNAs and diabetes, the circ_0043837 and circ_0001801 remained powerful and independent factors (**Figure 2C** and **Supplemental Table 3**). Furthermore, the LAA diagnostic model through the logistic regression, tested the regression model using the ROC. The circ_0043837 and circ_0001801 composite models showed an AUC of 0.825, its diagnostic efficacy was better than that of the single circ_0043837 (NRI=0.144) or circ_0001801 (NRI=0.097) (**Figure 2D**).

We assessed the possible severity of circRNAs with LAA. The National Institutes of Health Stroke Scale (NIHSS) is a recognized score scale corresponding to the extent and severity of the stroke (30). We performed a correlation analysis between DE circRNAs and NIHSS, in which circ_0043837 and circ_0001801 showed a negative statistically significant correlation (**Figure 2E**).

### Diagnostic Efficacy Comparison Between Exosomal CircRNAs and Plasma CircRNAs

Previous studies found that plasma circRNAs could be used as diagnostic markers for ischemic stroke (8). Recent studies showed that exosomes could protect circRNAs from degradation by biological enzymes (31). Thus, we performed differential expression assays of plasma circRNAs to verify whether exosomal circRNAs had better diagnostic efficacy.

**Figure 3:**
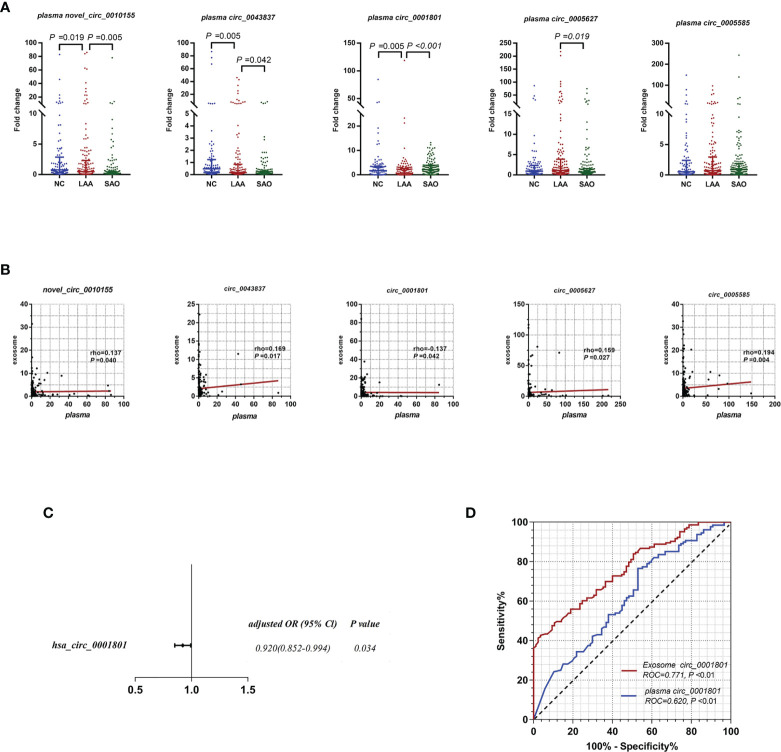
Diagnostic efficacy of LAA stroke comparison between exosome derived circRNA and plasma circRNAs. **(A)** Replication of plasma novel_circ_0010155, circ_0043837, circ_0001801, circ_0005627, and circ_0005585 in NC, LAA, and SAO groups. **(B)** Correlation between the assessment of novel_circ_0010155, circ_0043837, circ_0001801, circ_0005627, and circ_0005585 in peripheral blood-derived exosomes and plasma. **(C)** Multivariate logistic regression analysis for plasma circRNAs. **(D)** ROC curves of the exosomal and plasma circ_0001801.

As shown in **Figure 3A**, plasma novel_circ_0010155, circ_0043837, and circ_0001801 were DE between the LAA and the NC groups. The trend was the same as that of exosomal circRNAs. In addition, plasma novel_circ_0010155, circ_0043837, and circ_0001801, and circ_0005627 were significantly different between the SAO and LAA groups. However, plasma circ_0005585 was not statistically different among the three groups in the large sample replication.

We verified whether the expressions of plasma circRNAs and circulating exosomal circRNA were correlated. Plasma novel_circ_0010155, circ_0043837, circ_0005627, and circ_0005585 were positively correlated with exosomes, while circ_0001801 was negatively correlated (**Figure 3B**).

For plasma circRNAs, we included five plasma circRNAs for multivariate binary logistic regression, of which only circ_0001801 was associated with the LAA outcome variable (**Supplemental Table 4**). We further excluded the confounding factors of clinical indicators(diabetes), suggesting that plasma circ_0001801 was an independent factor (**Figure 3C**). We then constructed the diagnostic model, tested the regression model using the ROC, and evaluated the diagnostic efficacy of exosomes and plasma. For exosomal circ_0001801, the AUC of NC/LAA was 0.771. The AUC was greater than that of plasma circ_0001801 at 0.620, NRI (exo VS plasma) =0.183 (**Figure 3D**).

### Assessment Predictive Value Of Circulating Exosomal CircRNAs for LAA Plaque Rupture

**Figure 4:**
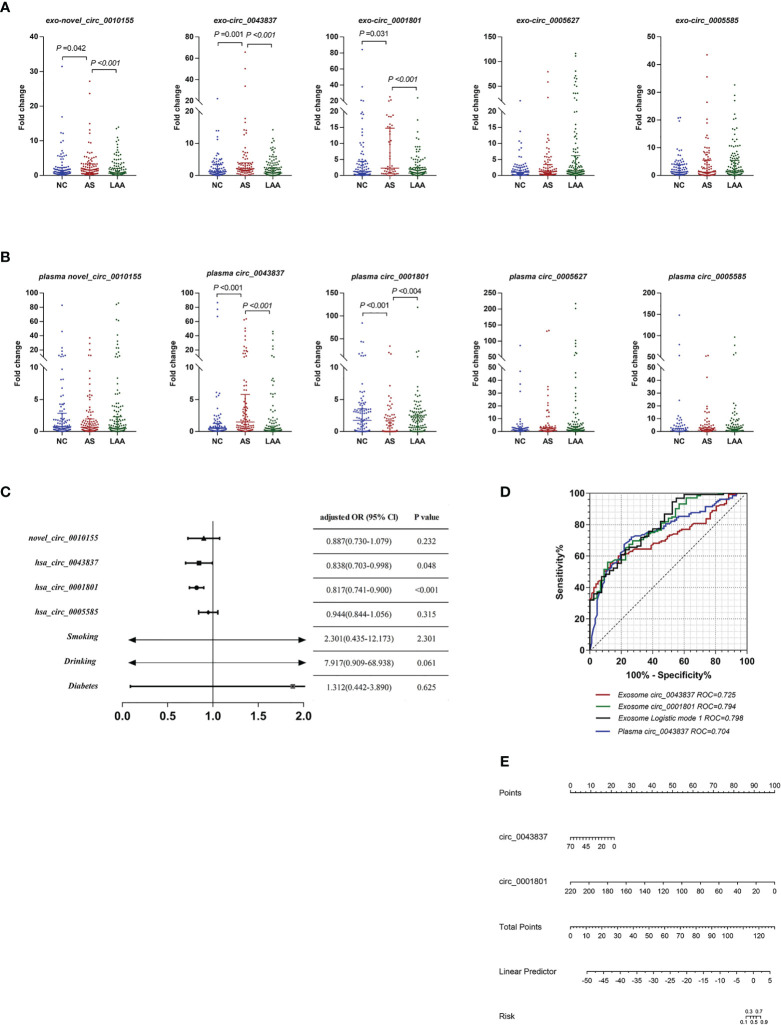
Diagnostic efficacy of plaque rupture of exosomal and plasma circRNAs. **(A)** Replication of exosomal circRNAs between NC, AS, and LAA group. **(B)** Replication of plasma circRNAs between NC, AS, and LAA group. **(C)** Multivariate logistic regression analysis for exosomal circRNAs and clinical. **(D)** ROC curves of the exosomal circ_0043837, circ_0001801, plasma circ_0043837, and logistic mode 1: Two-exo-circRNA of circ_0043837 and circ_0001801. **(E)** Nomograms to predict risk of plaque rupture.

Previous studies found that LAA was closely associated with unstable plaque rupture in AS (32). We further included the AS group for comparison with the LAA group to identify DE circRNAs that could predict plaque instability. First, we performed differential expression analysis of exosomal circRNAs and plasma circRNAs by RT-qPCR and assessed their diagnostic efficacy. Interestingly, there were also differences in exosomal novel_circ_0010155, circ_0043837, and circ_0001801 in AS compared with the NC and LAA groups (**Figure 4A**). Plasma circ_0043837 and circ_0001801 showed the same trend (**Figure 4B**).

Further we constructed a risk prediction model for plaque rupture. First, we included five circRNAs and the above clinical indicators for univariate logistic regression analysis, and multi-factor logistic regression analysis was performed for the meaningful risk factors (*P < 0.05*) among them (**Supplemental Table 5**). As shown in **Figure 4C**, where exosomal- circ_0043837 and circ_0001801 were independent risk factors. We analyzed the efficacy of circRNA alone and the composite model constructed by logistic regression by ROC, where the diagnostic efficacy of the composite model was superior to that of the single-factor index (**Figure 4D**). We also performed the same analysis for the five plasma circRNAs, where plasma circ_0043837 suggested an independent risk factor (**Supplemental Table 5**), where ROC analysis showed that exosomes had better diagnostic efficacy than plasma (**Figure 4D**). Subsequently, we included the above two exosomal circRNA constructs for clinical application in a nomogram, suggesting the risk of plaque rupture (**Figure 4E**).

## Discussion

In the present study, we evaluated exosomal circRNAs as diagnostic biomarkers for LAA stroke, which showed a superior diagnostic efficacy compared to plasma circRNAs. We also investigated its correlation with the severity of stroke. Meanwhile, our results provide evidence that exosomal circRNAs have potential predictive value of plaque rupture.

In the present study, we directly sequenced circulating exosomal circRNAs, in contrast to plasma RNA-seq reported previously. Circulating circRNAs are susceptible to degradation by biological enzymes; and the circRNAs are not specific. Recent studies found that exosomes, natural nanoparticle, being transporter-type vesicles, with a biofilm structure, protect circRNAs from biological enzymes and confer stability (12, 33, 34). Meanwhile, circRNAs are secreted by specific cells and are part of cellular targeting mediated by exosomal vesicles, which makes them reliable for the diagnosis of specific diseases (33, 35). Previous studies have shown that circulating exosomal miRNAs could be used as markers of early colon cancer with a better diagnostic efficacy than plasma (23). Therefore, in this study, we directly sequenced exosomal circRNAs and analyzed the DE circRNAs in the LAA to better identify biomarkers and conduct mechanistic studies.

As is a chronic inflammatory disease in which a large number of immune cells are involved. Macrophage uptake of ox-LDL to form macrophage-derived foam cells, which form fatty strips with T cells (36). We further selected exosomal circRNAs associated with immune and inflammatory processes for validation according to function enrichment. Novel_circ_0010155 is derived from ZNF91, which is distributed intracellularly and intranuclearly (37). It is involved in transcriptional regulation and highly expressed in T lymphocytes (38). circ_0043837 and circ_ 0005585 are associated with mitochondrial function. Mitochondrial damage affects macrophage function and is involved in the process of AS in previous studies (39). circ_0001801 and circ_0005627 are associated with gene regulation-related processes. circ_0001801 enrichment function is involved in the cellular protein modification process. In addition, circ_0005627 function is related to RNA modification. Therefore, based on functional enrichment and prediction, circRNAs such as novel_circ_0010155, circ_0043837, circ_0001801, circ_0005627, and circ_0005585 were selected for further validation.

Furthermore, we analyzed the DE circRNAs between the LAA and NC groups using small and large sample exosomal circRNA expression assays during biomarker screening. We included the SAO group (according to TOAST typing) in the NC group to account for possible circRNA interference caused by other symptoms such as stroke and to keep the false positive rate low in the validation of the LAA screening. Exosomal novel_circ_0010155, circ_0043837, circ_0001801, circ_0005627, and circ_0005585 were statistically different among the three groups in the small sample validation. circ_0043837, circ_0001801, circ_0005585, circ_0005627, and novel_circ_0010155 were significantly different before the LAA and NC groups in the expanded sample size assay. However, the latter two were not different between the LAA and SAO groups. This may suggest that the effect of stroke-related factors may have caused the differential expression of circ_0005627 and novel_circ_0010155 between the LAA and NC groups.

Previous studies suggested that exosomes could exert better protection against circRNAs (23, 31); therefore, in this study, we further examined plasma circRNA expression based on the study of differential expression of exosomal circRNAs and assessed the diagnostic efficacy of exosomal and plasma circRNAs. Our study found that there were statistically significant differences between the plasma LAA group circ_0043837, circ_0001801, and novel_circ_0010155 and the healthy NC and SAO groups. The same trend was observed in the exosomal circRNAs. We further constructed a diagnostic model of logistics based on differential expression and tested the diagnostic efficacy of exosomal and plasma circRNAs by means of the ROC. The exosomal AUC was higher than the plasma AUC and the NRI between exosomal and plasma circRNAs was above zero, thus suggesting that the diagnostic efficacy of exosomal circRNAs was better than that of plasma circRNAs. By combining circ_0043837 and circ_0001801 to construct the diagnostic model, the diagnostic efficacy of combined exosomal and plasma circRNAs was higher than that of a single index. Our study further suggested that exosomal circRNAs had greater diagnostic efficacy than circulating plasma circRNAs.

To further verify that circRNAs with diagnostic efficacy play a protective or facilitative role during LAA, we included NIHSS scores that were positively correlated with LAA disease severity. circ_0043837 and circ_0001801 were negatively correlated with the NIHSS scores. It was suggested that as the circRNA expression decreased, the NIHSS score and stroke severity increased. This suggested that circ_0043837 and circ_0001801 played a protective role in stroke.

In previous studies, intracranial and extracranial arterial plaque rupture and thrombosis were the main causes of ischemic stroke (40, 41). Therefore, prediction of atherosclerotic unstable plaque may help to prevent stroke. We included the AS group with > 50% intracranial and extracranial stenosis but without morbidity in our study, compared with the LAA brain infarction group with > 50% vascular stenosis to highlight the significance of plaque instability. circ_0043837 and circ_0001801 were DE in both exosomal and plasma circRNAs in the two groups. At the same time, we suggested that the above two circRNAs were independent risk factors for LAA by including five circRNAs and five clinical indicators (Smoking, drinking, hypertension, diabetes, and LDL), in turn, by univariate and multivariate regression analysis. These findings suggested that circ_0043837 and circ_0001801 were potential biomarkers for predicting plaque rupture. Furthermore, the impact of common clinical risk factors on the circRNAs and plaque rupture needs further investigation. In addition, the current study has limitations. These include the small sample size, the single-center sample collection, and the unavoidable selective bias. In addition to this, the plaques characteristics should be further defined with the imaging.

In conclusion, our study suggests that exosomal circRNAs can be used as diagnostic markers for stroke in LAA. We also found that exosomal circRNAs have better diagnostic efficacy than plasma circRNAs. We further identified that circRNAs, as biomarkers, could indicate plaque rupture. Our study provides an important basis for the diagnosis and prevention of LAA stroke.

## Data Availability Statement

The datasets presented in this study can be found in online repositories. The names of the repository/repositories and accession number(s) can be found below: NCBI Gene Expression Omnibus—GSE190869, GSE173719.

## Ethics Statement

Written informed consent was obtained from the individual(s) for the publication of any potentially identifiable images or data included in this article.

## Author Contributions

All authors read and approved the final version of the manuscript. XZ, XP were involved in the study design. QX, RH, HL, SZ, FZ collected the samples, performed the experiments and analyzed the data. QX, XZ completed the manuscript. All authors contributed to the article and approved the submitted version.

## Funding

We gratefully acknowledge financial support from the National Natural Science Foundation of China (No.82171299), and Natural Science Foundation of Shandong Province (ZR2020MH138).

## Conflict of Interest

The authors declare that the research was conducted in the absence of any commercial or financial relationships that could be construed as a potential conflict of interest.

## Publisher’s Note

All claims expressed in this article are solely those of the authors and do not necessarily represent those of their affiliated organizations, or those of the publisher, the editors and the reviewers. Any product that may be evaluated in this article, or claim that may be made by its manufacturer, is not guaranteed or endorsed by the publisher.
