# Supplementary material for: Circulating Exosomal circRNAs Contribute to Potential Diagnostic Value of Large Artery Atherosclerotic Stroke
Source: Front Immunol. 2022 Jan 13;12:830018. doi: 10.3389/fimmu.2021.830018 (PMC8792990; doi:10.3389/fimmu.2021.830018)
Supplement: Supplementary file 1 [file DataSheet_1.docx]

***Supplemental files***

**Supplemental Table S1**

**The primer sequences of qRT-PCR**

| RNAs | Primer Sequences (5’ → 3’) |
| --- | --- |
| novel_circ_0010155  (Junction_seq TAACCTTTTTTTTTTTTTTTTTGGCA  GGGTAGGTGAGAGTGAATACAACAGACGAC) | Forward CAGGGCCCAGGGAAATGAGA  Reverse GCCTCTCATCCTTGTCGTCTGT |
| hsa_circ_0043837 | Forward TGGTCCTTCGCCGTCAGTAT  Reverse ACAGACTCAGGCTGACTCCA |
| hsa_circ_0001801 | Forward GAACCGGATATTTAAGTACAATGGTGG  Reverse TCACAGCTCCTCCCATGATAGT |
| hsa_circ_0005627 | Forward GAGTCATTCACGCCATTGCT  Reverse GCTGCTCTCTTCTTTTGCGT |
| hsa_circ_0005585 | Forward AAACATGTGACGCGCTCCAG  Reverse CCAGCTGTTGAAGTGGTGCC |

**Supplemental Table S2.**

**Clinical characteristics of LAA patients and control subjects**

| Variables | NC groups | LAA groups | SAO groups | AS groups |
| --- | --- | --- | --- | --- |
| Demographic  Total, n | 149 | 196 | 170 | 106 |
| Sex (Female, %) | 72 (48.32%) | 92 (46.94%) | 81 (47.65%) | 54 (50.94%) |
| Ages (years) | 64.05 ± 1.11 | 62.94 ± 0.88 | 62.81 ± 0.10499 | 65.16 ± 1.01 |
| Clinical |  |  |  |  |
| Smoking (n, %) | 47 (31.54%) | 63 (32.14%) | 55 (32.35%) | 32 (30.19%) |
| Drinking (n, %) | 44 (29.53%) | 61 (31.12%) | 51 (30.00%) | 31 (29.25%) |
| Hypertension (n, %) | 82 (55.03%) | 122 (62.24%) | 105 (61.76%) | 65 (61.32%) |
| Diabetes (n, %) | 28 (18.79%) | 53 (27.04%) | 43 (25.29%) | 21 (19.81%) |
| NIHSS score | 0 | 5.65 ± 0.44 ^*^ | 3.06 ± 0.22 ^*#^ | 0 |
| Laboratory |  |  |  |  |
| TG (mmol/L) | 1.80 ± 0.14 | 1.42 ± 0.09^*^ | 1.54 ± 0.08 | 1.58 ± 0.16 |
| TC (mmol/L) | 4.64 ± 0.10 | 4.27 ± 0.10^*^ | 4.44 ± 0.09 | 4.26 ± 0.23 |
| LDL (mmol/L) | 2.63 ± 0.08 | 2.56 ± 0.08 | 2.71 ± 0.06 | 2.57 ± 0.14 |
| HDL (mmol/L) | 1.35 ± 0.03 | 1.24 ± 0.03 | 1.25 ± 0.03 | 1.17 ± 0.06 |
| Lipoprotein a (mmol/L) | 204.30 ± 17.46 | 234.02 ± 17.54 | - 1. ± 15.23 | 256.07± 36.64 |

* *P* <0.05, compared with NC group, # *P* <0.05, compared with LAA group

**Supplemental Table S3**

**Logistic regression analysis for exo-circRNAs of LAA patients and control subjects**

|  | **crude OR** | | | **adjusted OR** | | |
| --- | --- | --- | --- | --- | --- | --- |
|  | **OR** | **(95% Cl)** | ***P* value** | **OR** | **(95% Cl)** | ***P* value** |
| novel_circ_0010155 | 0.920 | (0.838-1.009) | 0.077 |  |  |  |
| hsa_circ_0043837 | 0.679 | (0.561-0.821) | <0.001 | 0.623 | (0.471-0.823) | 0.001 |
| hsa_circ_0001801 | 0.809 | (0.735-0.891) | <0.001 | 0.788 | (0.696-0.891) | <0.001 |
| hsa_circ_0005627 | 1.078 | (1.012-1.149) | 0.021 | 1.065 | (0.991-1.144) | 0.085 |
| hsa_circ_0005585 | 0.961 | (0.890-1.037) | 0.307 |  |  |  |
| Smoking | 1.525 | (0.842-2.762) | 0.164 |  |  |  |
| Drinking | 1.716 | (0.935-3.151) | 0.081 |  |  |  |
| Hypertension | 1.633 | (0.955-2.791) | 0.073 |  |  |  |
| Diabetes | 1.959 | (1.050-3.654) | 0.035 | 1.469 | (0.578-3.736) | 0.419 |
| LDL (mmol/L) | 0.906 | (0.665-1.235) | 0.532 |  |  |  |

**Supplemental Table S4**

**Logistic regression analysis for plasma circRNAs of LAA patients and control subjects**

|  | **crude OR** | | | **adjusted OR** | | |
| --- | --- | --- | --- | --- | --- | --- |
|  | **OR** | **(95% Cl)** | ***P* value** | **OR** | **(95% Cl)** | ***P* value** |
| novel_circ_0010155 | 1.005 | (0.983-1.028) | 0.633 |  |  |  |
| hsa_circ_0043837 | 0.967 | (0.926-1,011) | 0.137 |  |  |  |
| hsa_circ_0001801 | 0.917 | (0.849-0.991) | 0.028 | 0.920 | (0.852-0.994) | 0.034 |
| hsa_circ_0005627 | 1.018 | (0.097-1.040) | 0.094 |  |  |  |
| hsa_circ_0005585 | 0.993 | (0.975-1.011) | 0.307 |  |  |  |

**Supplemental Table S5**

**Logistic regression analysis for circRNAs of AS subjects and LAA patients.**

|  | **crude OR** | | | **adjusted OR** | | |
| --- | --- | --- | --- | --- | --- | --- |
|  | **OR** | **(95% Cl)** | ***P* value** | **OR** | **(95% Cl)** | ***P* value** |
| **Exosome** |  |  |  |  |  |  |
| novel_circ_0010155 | 0.903 | (0.828-0.985) | 0.021 | 0.887 | (0.730-1.079) | 0.232 |
| circ_0043837 | 0.856 | (0.781-0.939) | 0.001 | 0.838 | (0.703-0.998) | 0.048 |
| circ_0001801 | 0.818 | (0.756-0.86) | <0.001 | 0.817 | (0.741-0.900) | <0.001 |
| circ_0005627 | 1.022 | (0.999-1.046) | 0.062 |  |  |  |
| circ_0005585 | 0.933 | (0.877-0.991) | 0.025 | 0.944 | (0.844-1.056) | 0.315 |
| **Plasma** |  |  |  |  |  |  |
| novel_circ_0010155 | 1.022 | (0.990-1.055) | 0.182 |  |  |  |
| circ_0043837 | 0.951 | (0.918-0.986) | 0.006 | 0.951 | (0.918-0.985) | 0.005 |
| circ_0001801 | 1.021 | (0.969-1.075) | 0.437 |  |  |  |
| circ_0005627 | 1.006 | (0.994-1.018) | 0.318 |  |  |  |
| circ_0005585 | 0.996 | (0.972-1.022) | 0.772 |  |  |  |
| **Clinical** |  |  |  |  |  |  |
| Smoking | 2.253 | (1.168-4.348) | 0.015 | 2.301 | (0.435-12.173) | 2.301 |
| Drinking | 4.886 | (2.183-10.936) | <0.001 | 7.917 | (0.909-68.938) | 0.061 |
| Hypertension | 0.826 | (0.466-1.464) | 0.512 |  |  |  |
| Diabetes | 1.919 | (1.029-3.578) | 0.040 | 1.312 | (0.442-3.890) | 0.625 |
| LDL (mmol/L) | 0.986 | (0.683-1.424) | 0.940 |  |  |  |


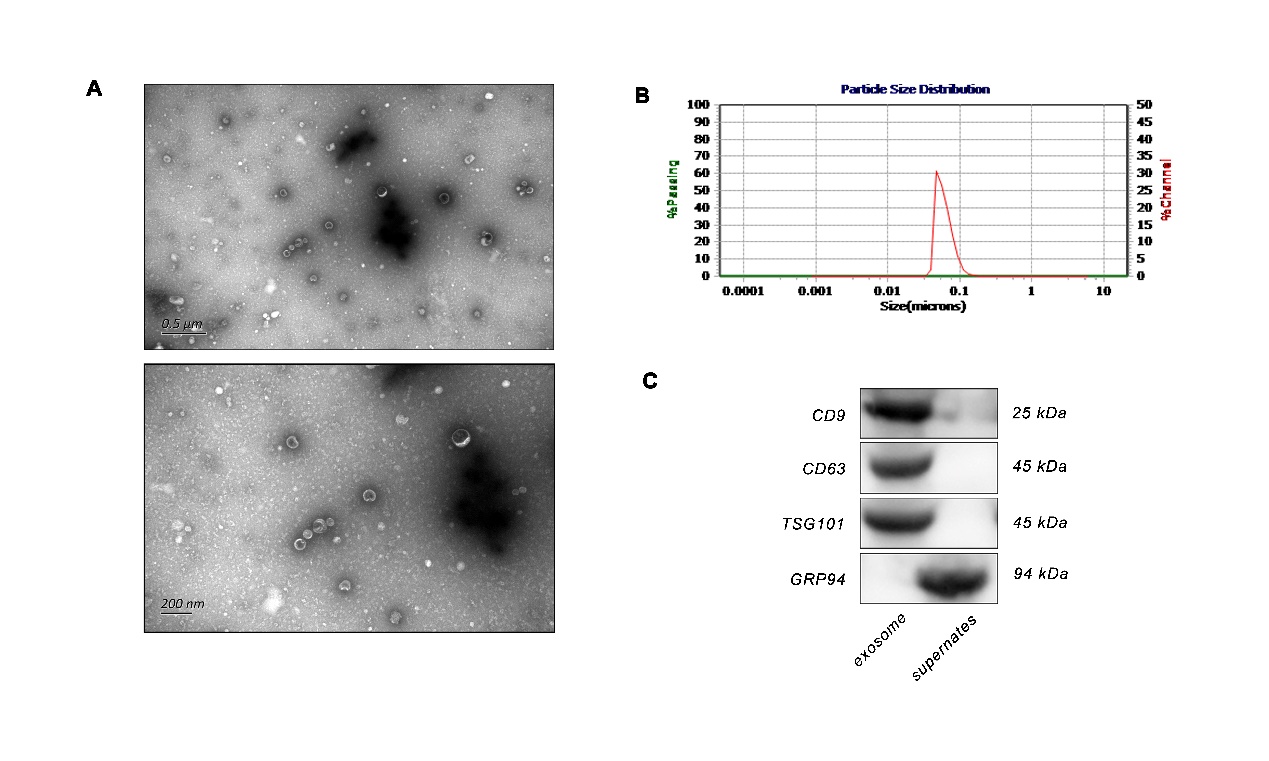


**Supplemental Figure 1. Characterization of circulating exosomes. (A)** Electron micrograph of the exosomes. **(B)** NTA of the exosomes size distribution. **(C)** Western blot of the exosomes and supernates protein markers.


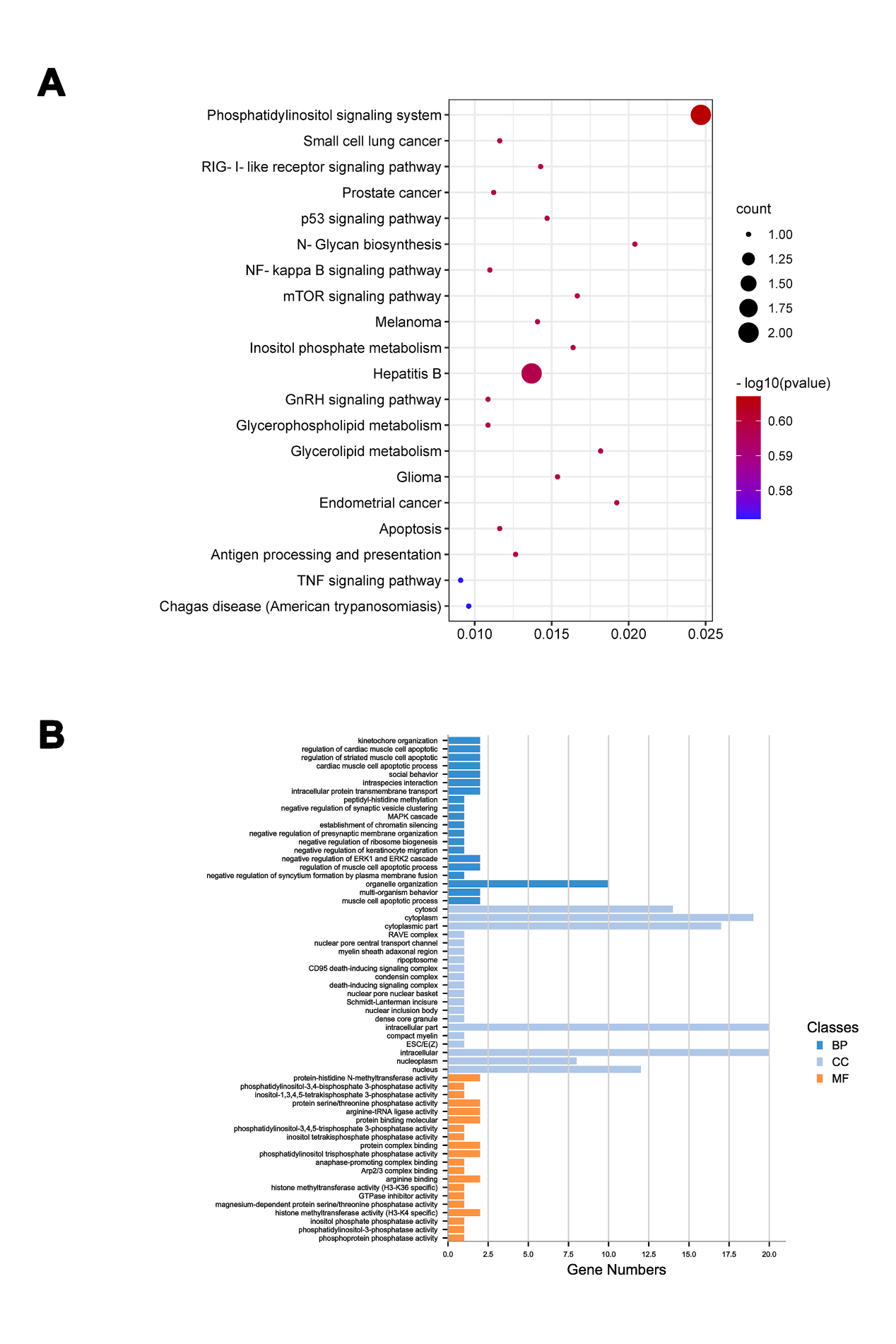


**Supplemental Figure 2.** **Functional enrichment of exosome-circRNAs from RNA-Seq. (A)** Functional enrichment map of the KEGG pathway with differentially expressed circRNAs. **(B)** GO functional enrichment map of differentially expressed circRNAs.
